# Chronic iron deposit and left ventricular remodeling in reperfused STEMI patients

**DOI:** 10.1186/1532-429X-18-S1-P230

**Published:** 2016-01-27

**Authors:** Heerajnarain Bulluck, Steven K White, Stefania Rosmini, Amna Abdel-Gadir, Anish N Bhuva, Thomas A Treibel, Marianna Fontana, Patricia Reant, Manish Ramlall, Ashraf Hamarneh, Alex Sirker, Anna S Herrey, Charlotte Manisty, Peter Kellman, James Moon, Derek J Hausenloy

**Affiliations:** 1 grid.83440.3b0000000121901201The Hatter Cardiovascular Institute, University College London, London, United Kingdom; 2 Cardiac Imaging, Barts Heart Centre, London, United Kingdom; 3 Barts heart Centre, London, United Kingdom; 4 grid.94365.3d0000000122975165NIH, Bethesda, CA USA

## Background

After reperfused ST-segment elevation myocardial infarction (STEMI), infarct zone microvascular obstruction (MVO) and intramyocardial hemorrhage are associated with left ventricular (LV) remodeling. We wanted to understand what happened to areas of haemorrhage and observe its resolution/persistence and any role it may play in remodeling.

## Methods

48 STEMI patients underwent CMR imaging at 1.5T (Siemens Avanto) at 4 ± 2 days post-PPCI and 40 completed a follow-up scan at 5 ± 2 months. Left ventricular (LV) short-axis native T1 (MOLLI), T2 and T2* maps were acquired. MVO was indicated by a hypo-intense core on LGE images. A hypo-intense core on T2*-maps with a T2*<20 ms was used for IMH (acutely) or chronic iron deposit (at follow-up). Mean segmental T2 and T1 values were obtained using CVI42 (Calgary, Canada). LV remodeling was defined as a 20% increase in LV end-diastolic volume on the follow-up scans.

## Results

*Acute scan*

**Figure 1:**
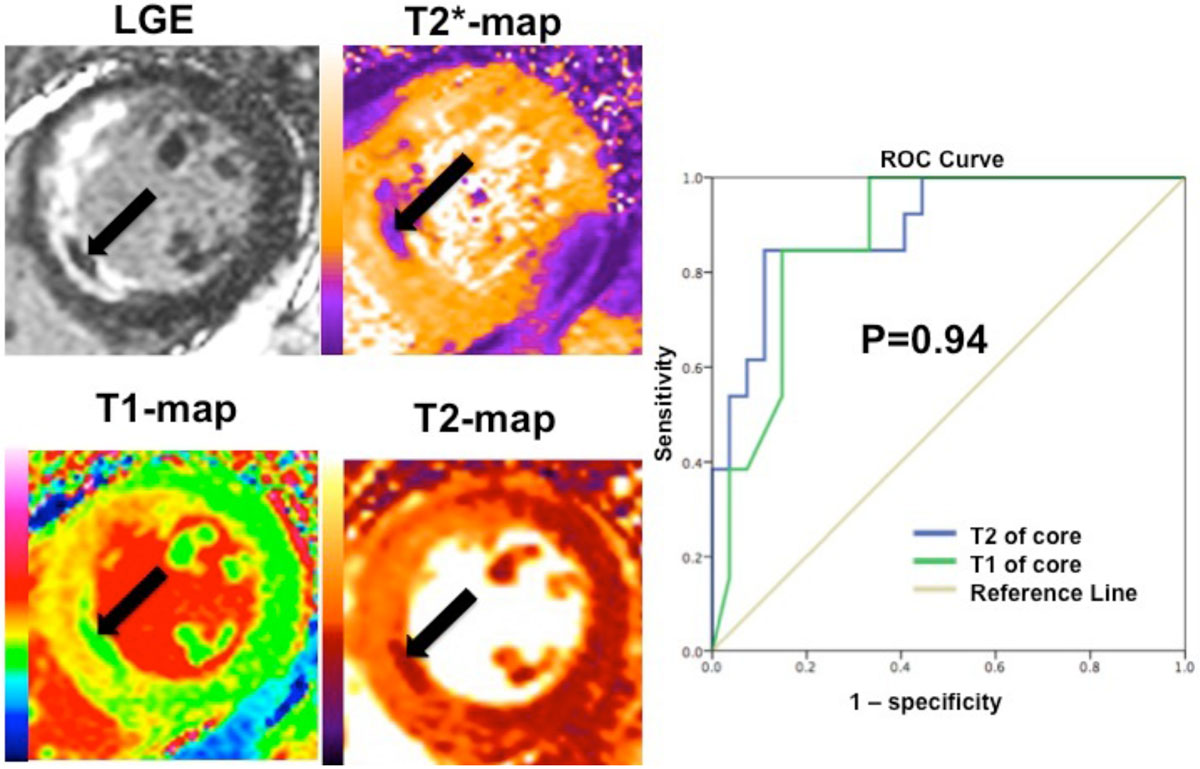
Matching LV short-axis images of LGE, T2*, T1, T2-maps in a patient with MVO (arrows indicate MVO with IMH). ROC curves of T1 and T2 values in the hypo-intense core for detecting IMH.

MVO was present in 63% patients. T2* maps was used as the reference standard (17% excluded due to motion and breathing artifacts rendering then uninterpretable). T1 and T2-mapping performed equally well in detecting the presence of IMH on the acute scan (T1: AUC 0.86 [95%CI 0.72-0.99], T2: AUC 0.86 [95%CI 0.74-0.99]; P=0.94)(Figure 1). 29/30(95%) patients with MVO had evidence of IMH (T2*<20 ms).

*Follow scan*

**Figure 2:**
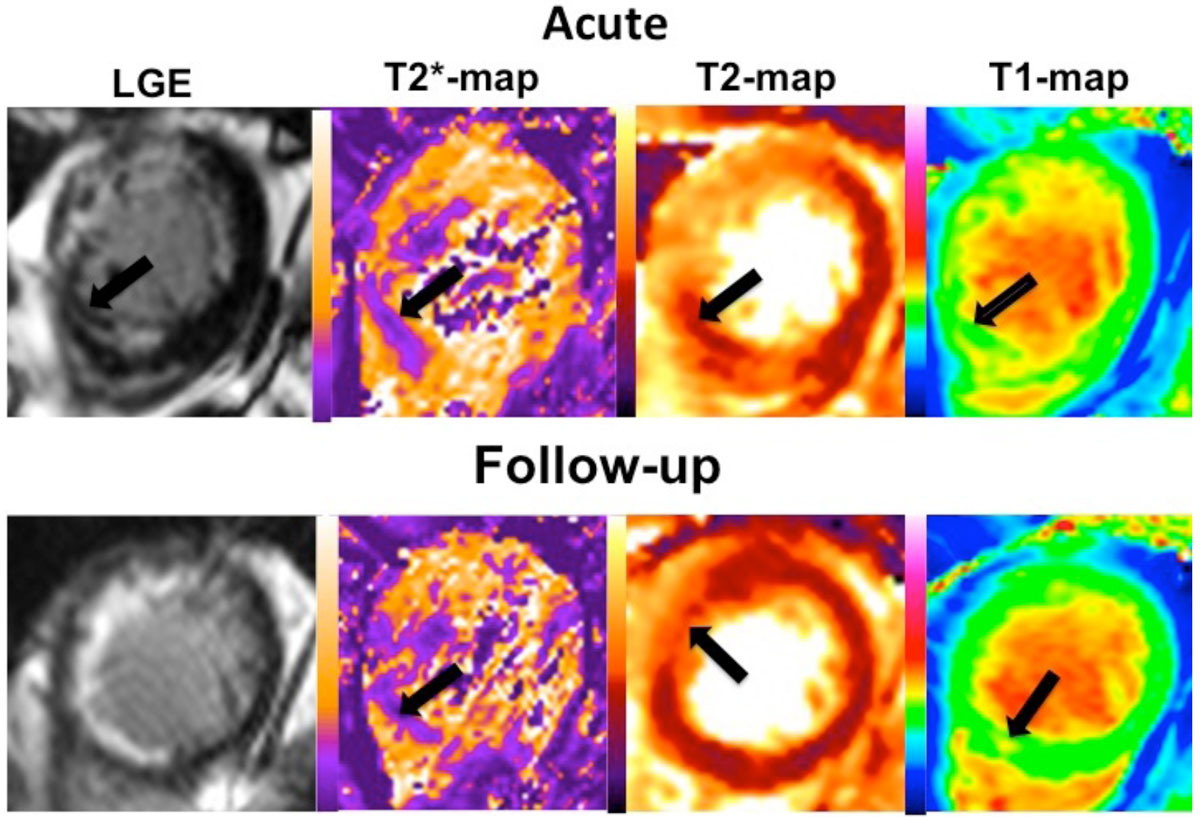
LGE, T2*, T2 and T1 maps in a patient with an anterior STEMI. Black arrows show the presence of MVO with IMH. The MVO has disappeared on the follow-up scan leaving chronic iron deposition as indicated by a persistent hypo-intense core on T2* scan and areas of normal T2 values (black arrow). Adjacent the areas of iron deposition are areas of high T2, which may indicate a reactive inflammatory response.

8/40(20%) patients had evidence of LV remodeling on the follow-up scan. All patients who developed LV remodeling had MVO and IMH acutely, compared to 60% in patients without LV remodeling (P=0.04). 37% of T2* maps were excluded on the follow-up scan. 13/15(87%) of patients had evidence of chronic iron deposition within the infarct core (T2*15 ± 2ms). In these patients, T1 and T2 values were elevated within the areas of late gadolinium enhancement (Figure 2) and were higher than those without iron deposit.

## Conclusions

T1 and T2-mapping can detect hemorrhage.

Acute hemorrhage becomes chronic and is still detectable at 5 months

Infarct T2 normalizes usually by 3-6 months in most patients - but around chronic iron the T2 remains high - suggesting the iron may be a source of ongoing inflammation.

